# Assessing Accuracy of Genomic Predictions for Resistance to Infectious Hematopoietic Necrosis Virus With Progeny Testing of Selection Candidates in a Commercial Rainbow Trout Breeding Population

**DOI:** 10.3389/fvets.2020.590048

**Published:** 2020-11-05

**Authors:** Roger L. Vallejo, Breno O. Fragomeni, Hao Cheng, Guangtu Gao, Roseanna L. Long, Kristy L. Shewbridge, John R. MacMillan, Richard Towner, Yniv Palti

**Affiliations:** ^1^National Center for Cool and Cold Water Aquaculture, Agricultural Research Service, United States Department of Agriculture, Kearneysville, WV, United States; ^2^Department of Animal Science, University of Connecticut, Storrs, CT, United States; ^3^Department of Animal Science, University of California, Davis, Davis, CA, United States; ^4^Clear Springs Foods Inc., Research Division, Buhl, ID, United States; ^5^Gen Tec Consulting, Payette, ID, United States

**Keywords:** bayesian multiple regression, disease resistance, genomic selection, infectious hematopoietic necrosis virus, rainbow trout, single-step GBLUP

## Abstract

Infectious hematopoietic necrosis (IHN) is an economically important disease of salmonid fish caused by the IHN virus (IHNV). Under industrial aquaculture settings, IHNV can cause substantial mortality and losses. Actually, there is no confirmed and cost-effective method for IHNV control. Clear Springs Foods, Inc. has been performing family-based selective breeding to increase genetic resistance to IHNV in their rainbow trout breeding program. In an earlier study, we used siblings cross-validation to estimate the accuracy of genomic prediction (GP) for IHNV resistance in this breeding population. In the present report, we used empirical progeny testing data to evaluate whether genomic selection (GS) can improve the accuracy of breeding value predictions over traditional pedigree-based best linear unbiased predictions (PBLUP). We found that the GP accuracy with single-step GBLUP (ssGBLUP) outperformed PBLUP by 15% (from 0.33 to 0.38). Furthermore, we found that ssGBLUP had higher GP accuracy than weighted ssGBLUP (wssGBLUP) and single-step Bayesian multiple regression (ssBMR) models with BayesB and BayesC priors which supports our previous findings that the underlying liability of genetic resistance against IHNV in this breeding population might be polygenic. Our results show that GS can be more effective than either the traditional pedigree-based PBLUP model or the marker-assisted selection approach for improving genetic resistance against IHNV in this commercial rainbow trout population.

## Introduction

Infectious hematopoietic necrosis (IHN) is an important disease of salmonid fish that is caused by IHN virus (IHNV), which is a single-stranded negative-sense RNA rhabdovirus (1). IHNV is endemic to the Pacific Northwest in North America (2) and has spread through continental Europe, China, and Japan (3–5). IHNV is infectious to Pacific salmon and trout *(Oncorhynchus* spp.), as well as to Atlantic salmon (*Salmo salar)* (2), and can cause significant mortality and losses at nearly all stages of production in commercial aquaculture settings (6–8). Currently, unfortunately, there is no confirmed and cost-effective method for IHNV prevention or treatment. Therefore, the development of rainbow trout strains with genetic resistance to IHNV is needed for improving animal well-being and decreasing economic losses that are inflicted by this infectious viral induced disease to food aquaculture production (8).

The additive genetic basis for IHNV resistance is evident from the moderate estimates of heritability for IHNV binary survival status *(h*^2^ = 0.23–0.55) and survival days to death (*h*^2^ = 0.02–0.20) in a steelhead trout population (2). Recently, we also found moderate estimates of heritability for IHNV binary survival status *(h*^2^ = 0.25–0.28) and survival days to death *(h*^2^ = 0.23–0.33) in a commercial rainbow trout breeding population (8). These reports suggest that rainbow trout strains with genetic resistance against IHNV can be developed by means of traditional family-based selective breeding. However, up to now, the development of strains with enhanced resistance to IHNV in rainbow trout has been limited to traditional pedigree-based selection. The process of selecting and developing IHNV-resistant strains is further complicated due to the high genetic variability of the IHN virus as revealed by phylogenetic and nucleotide sequence analyses of 84 IHNV isolates (9). Nevertheless, selective breeding of a rainbow trout strain for resistance to IHNV has been conducted at the Clear Springs Foods Inc. (CSF) breeding program since the year 2000 (10), and the selection differential for resistance to IHNV has been on average 10% for the last eight generations between 2000 and 2016 (8).

Few quantitative trait loci (QTL) mapping research have been previously conducted to detect genetic polymorphisms linked with IHNV resistance and to determine the genetic basis of IHNV resistance in rainbow trout populations. A number of moderate-large-effect QTL associated with IHNV resistance were detected on 12 rainbow trout chromosomes using linkage analysis (3, 11–13) and genome-wide association studies (GWAS) (10). However, these earlier QTL mapping studies had several experimental limitations which we described and discussed elsewhere (8). To this end, we have recently performed GWAS for IHNV resistance using a 57K SNP panel and multiple regression single-step methods and found that the inheritance of resistance to IHNV in the Clear Springs breeding population is controlled by up to 10 small-moderate effect QTL (explained genetic variance = 2.0–8.8%) and large-unknown number of minute effect loci (8).

There is uncertainty on the best computational algorithm when using multiple regression based models in GWAS and genomic selection (GS) studies because the underlying genetic bases of a complex trait and the population structure can have major impact on the statistical power to detect marker effects and on the accuracy of genomic prediction (GP) (8). Therefore, it is recommended to compare the results from the best existing computational GWAS and GP methods when elucidating the genetic architecture of a complex disease and performing GP for the first time in a population (8).

When using multiple regression based GWAS and GP models that fit all single nucleotide polymorphisms (SNPs) with high quality genotypes, the genomic best linear unbiased prediction (GBLUP) method assumes that all SNPs have a non-zero contribution to the variance of the studied trait, with equal variance for each SNP, and that the distribution of the SNP effects follows a normal distribution (14–16). In addition, the single-step GBLUP (ssGBLUP) approach was developed, which combines the pedigree-based (**A**) and genomic relationship (**G**) matrices into the **H** relationship matrix (17, 18). On the other hand, the Bayesian variable selection model assumes that the trait genetic variance is explained by a relatively small number of loci, each with a small-moderate or large effect (14, 19–23). Based on the underlying assumptions of these models, the GBLUP model is expected not to perform as well as the Bayesian variable selection model when the trait genetic architecture is not mainly polygenic. For that reason, the GBLUP method was extended to the weighted ssGBLUP (wssGBLUP) method. The wssGBLUP mimics the Bayesian variable selection model by fitting all SNPs in the multiple regression model but assigning differential weights to the SNPs based on the variance of each SNP effect (8, 24). Lately, Bayesian variable selection models that use a single-step approach have been developed (25–28), including the single-step Bayesian multiple regression (ssBMR) method (8, 25, 28).

Assessment of the accuracy and bias of GP using progeny testing data is preferred over cross-validation (CV) analysis due to these reasons: First and foremost, there is a practical breeding purpose for GS, which is the actual de-facto improvement from selective breeding by passing over genetic variants or alleles associated with superior performance from parents to their offspring. Second, the CV analysis is unreliable because it is highly stochastic, and the GP accuracy is impacted by the magnitude of the estimated heritability. Third, it has been shown that the correlation of mid-parent genomic estimated breeding value (GEBV) with the mean progeny performance for each progeny testing family is a reliable estimator of the accuracy of the predicted breeding value (BV) (29, 30). Fourth, the bias of BV predictions for binary phenotypes (i.e., disease survival STATUS) when using CV analysis is incorrect (8) because all the binary observations within a class of variables are identical (i.e., each animal has one binary survival phenotype record of either 0 or 1) (31). With progeny testing data this drawback is circumvented by calculating the accuracy and bias of BV predictions using progeny performance data per evaluated progeny testing family (e.g., average survival rate) (8, 23, 32).

The development of multiple-regression based GWAS and GS methods, along with the manufacturing of the rainbow trout 57K SNP array (33) have provided the critical tools to perform whole genome-enabled selection for resistance against IHNV in rainbow trout. Recently, we estimated GP accuracies in the range of 0.30–0.39 for IHNV resistance in the CSF rainbow trout breeding population using simulation-based CV analysis (8). In the current study, we aimed to validate those estimated GP accuracies using empirical progeny performance data. Thus, the specific objectives of the study were to (1) evaluate the accuracy and bias of BV predictions for IHNV resistance using empirical data from progeny testing evaluations, (2) compare the accuracy and bias of BV predictions estimated with multiple regression single-step methods (ssGBLUP, wssGBLUP and ssBMR) and traditional pedigree-based PBLUP, (3) determine the extent of linkage disequilibrium (LD) and the effective population size (*N*_*e*_) in the CSF population, and (4) assess the impact of relatedness between training and testing animals on the GP accuracy in commercial rainbow trout breeding populations.

## Materials and Methods

### Ethics Statement

This study used rainbow trout fin clips collected after controlled exposure to IHNV as part of a selective breeding program at the CSF research facility. As farm animals used in a commercial breeding program, these fish are exempted from regulation under the U.S. Animal Welfare Act and therefore not subject to oversight by an Institutional Animal Care and Use Committee or other such ethics committee. This exemption is defined in U.S. Code title 7, chapter 54, section 2,132 g. However, experimentation and handling were conducted in accordance with U.S. government principals for the use and care of vertebrate animals used in testing, research, and training, which includes provisions to minimize animal suffering. Specific measures for amelioration of animal suffering during the fish pathogen testing included minimization of handling, maintenance of optimal water temperature, and oxygen saturation, and the fish were fed a standard fish meal diet to satiation daily. Fish near death from severe symptoms of infection during the observation period were removed and terminated (by immersion in a lethal dose of MS222) before collection of fin tissue to minimize suffering. After the 3-week observation period, surviving fish were terminated by immersion in a lethal dose of MS222 before sampling and disposal.

### Fish Growing and IHNV Disease Challenge

Samples were collected from disease-naïve parents and their disease challenged offspring fish in brood years 2014 and 2016, respectively, by staff at the CSF research facility in Buhl, Idaho, and processed following already described procedures (8). Briefly, healthy fish from the previous generation were artificially spawned to produce fertilized eggs from 104 families of year-class (YC) 2016. Fin tissue samples from each parent fish were collected at the time of spawning. The offspring were grown to ~1 g (62 days post-fertilization) and 50 fish per family were selected randomly for disease challenge and were infected with IHNV by immersion into a volume of water equivalent to 10x the total body weight of the fish in g containing 10,000 plaque-forming units of IHNV per mL for 1 h (IHNV isolate 220-90). After exposure, the fish were moved to 19-L tanks by family (50 fish/family/tank), because young and small fish cannot be labeled individually, and monitored for a 21-day period, with mortality recorded daily. Fin tissue samples were collected from mortalities during the 21-day monitoring period and survivor samples were taken at the end of the challenge. Fin clips from all fish (mortalities and survivors) were individually kept in 95% ethanol until DNA was isolated using published protocols (34).

### Training and Testing Sample for GP

The training sample of this GP study comprised 104 pedigreed full-sib (FS) families from YC 2016 of the CSF commercial breeding company. The 104 families included 19 paternal half-sib (HS) families and two maternal HS families, and they were generated using 81 sires and 101 dams (Table 1). Sixty-three families were made by mating each of 63 sires with a single dam. Among the families that were made by mating a sire with multiple dams: 17 sires were mated with two dams, one sire was mated with three dams, and another sire was mated with four dams. Among the families that were produced by mating a dam with multiple sires: two dams were mated with two sires. These YC 2016 families represented a commercial nucleus breeding population that was undergoing intensive selection for growth and IHNV resistance for the past eight generations. The fish were evaluated for IHNV resistance in the laboratory challenge, with one tank per family with an initial stocking of 50 fish per tank. The 104 families were evaluated by groups of 7–10 families at 11 challenge dates. After the IHNV challenge, IHNV resistance phenotype records on *N* = 5,191 fish were collected. From the total 104 training sample families, 100 FS families were genotyped with an average of 14 offspring fish per family (range 9–30 fish) for a total of *N* = 1,449 genotyped training offspring fish.

**Table 1:** Experimental variables of genomic selection for IHNV resistance^a^.

**Experimental variable**	**Training**	**Testing**	**Progeny performance**
Phenotyped FS families	104	Na^b^	62
Phenotyped sire-HS families	19	Na	10
Phenotyped dam-HS families	2	Na	0
Number of sires	81	Na	52
Number of dams	101	Na	62
MEAN-PHENOTYPED offspring per family	50	Na	100
MIN-PHENOTYPED offspring per family	43	Na	99
MAX-PHENOTYPED offspring per family	52	Na	100
TOTAL phenotyped fish	5,191	Na	6,198
Genotyped FS families	100	35	Na
Genotyped sire-HS families	18	4	Na
Genotyped dam-HS families	2	2	Na
Families GENOTYPED in both TRAINING & TESTING sets	35	35	Na
Families GENOTYPED only in TRAINING set	65	Na	Na
Families GENOTYPED only in TESTING set	Na	0	Na
MEAN-GENOTYPED offspring per family	14	4	Na
MIN-GENOTYPED offspring per family	9	1	Na
MAX-GENOTYPED offspring per family	30	8	Na
Genotyped parents	51	Na	Na
Genotyped offspring	1,449	124	Na
TOTAL genotyped fish	1,500	124	Na
Number of pedigree records	6,693	Na	Na

a *Genomic selection for infectious hematopoietic necrosis virus (IHNV) resistance conducted in the Clear Springs Foods, Inc. year-class 2016 breeding population*.

b *Na indicates either non-available or non-needed data cell*.

The testing sample of this GP study comprised 35 FS families which were part of the genotyped 100 training FS families. Specifically, from the 100 families that were genotyped as part of the training sample, 35 families were also included in the testing sample. Thus, there were 35 families that contributed offspring fish to both the training and the testing samples. From each testing family, an average of 4 offspring fish were genotyped (range 1–8 fish) for a total of *N* =124 genotyped testing offspring fish (Table 1).

### Progeny Testing Families

In this GS study, 62 progeny testing families (PTF) from the YC 2018 were IHNV challenged to assess the accuracy of the predicted breeding values (BVs) for fish included in the testing sample (Table 1). The 62 PTFs were created using 52 sires and 62 dams; and these 114 breeders were taken from the testing genotyped sample (i.e., these breeders are offspring of the 35 families included in the testing sample). A sample of ~100 fish from each PTF was IHNV challenged to generate IHNV disease survival records from a total of *N* = 6,198 fish.

### IHNV Resistance Phenotypes

We had a binary survival status (STATUS) record for each evaluated fish. The resistance phenotype STATUS had two categories: 1 for fish that died during the 21 days post challenge evaluation period; and 2 for fish that survived for the duration of the challenge. The binary records of disease survival STATUS were analyzed using animal threshold models described below.

### SNP Genotyping

The fish sampled from the CSF population were genotyped by a commercial service provider (AKESOgen, Norcross, GA and RUCDR, Rutgers University, Piscataway, NJ) using the Rainbow Trout Axiom 57K SNP array (Chip) following previously described procedures (8, 33). We randomly sampled survivor offspring and early dying offspring per family with an average of 14 fish per family (range of 9–30) from a total of 100 training families (offspring *N* = 1,449) for SNP genotyping. We also genotyped all the sires from which fin clips were available (*N* = 51). The dams were not sampled. As part of the testing sample, we randomly sampled an average of four offspring fish per family (range 1–8) from 35 families out of the 100 training families (offspring *N* = 124) for SNP genotyping (Table 1). The quality control (QC) pipeline procedures applied to the Chip-SNP genotype data was described elsewhere (23). Briefly, the QC pipeline discarded the SNPs that showed a significant distortion from the expected Mendelian segregation in each FS family (Bonferroni adjusted to *P* < 0.05) and also removed offspring fish that did not have matching genotypes with the parents given in the pedigree (i.e., that did not pass the pedigree check). After this initial data QC, we had genotype data for 42,045 SNPs in the raw Chip genotype dataset.

Before GS analyses, the raw marker genotype dataset was further QC filtered using computer algorithms from the software BLUPF90 (35) and procedures already described (8). Briefly, the QC retained SNPs with a genotype calling rate higher than 0.90, minor allele frequency higher than 0.05, and with departures from Hardy-Weinberg equilibrium lower than 0.15, based on the difference between expected and observed frequency of heterozygotes. Parent-progeny pairs were tested for discrepant homozygous SNPs, and SNPs with a conflict rate higher than 1% were removed from the dataset. Next, we determined the physical map location (GenBank Assembly Accession GCA_002163495.1) (36, 37) of each of the QC filtered SNPs and those that did not have a physical map location were discarded. After this final data QC, we had data on 34,640 genotyped SNPs and 1624 genotyped fish (1,449 training offspring and 124 testing offspring from YC 2016 and 51 sires from YC 2014) for GS analysis (Tables 1, 2).

**Table 2:** Estimated genetic parameters for IHNV resistance in a commercial rainbow trout breeding population^a^.

**Method^b^**	**Phenotyped^c^**		**Genotyped**			**Genetic parameter^f^**			
	**Families**	**Fish**	**Families**	**Fish**	**SNPs**	$\sigma_{a}^{2}$	$\sigma_{f/d}^{2}$	$\sigma_{e}^{2}$	***h*^2^**
PBLUP	104	5,191	Na^d^	Na	Na	0.950 ± 0.518	0.231 ± 0.88	1.002 ± 0.029	0.25 ± 0.12
ssGBLUP^e^	104	5,191	100	1,624^g^	34,640	0.657 ± 0.156	0.191 ± 0.063	1.001 ± 0.028	0.22 ± 0.06

a *Clear Springs Foods, Inc. year-class 2016 rainbow trout breeding population*.

b *Variance components for infectious hematopoietic necrosis virus (IHNV) resistance were estimated using pedigree-based BLUP (PBLUP) and PBLUP with genomics data (ssGBLUP)*.

c *IHNV resistance phenotype: binary fish survival status (STATUS) after IHNV challenge*.

d *Na indicates that data are not available; the PBLUP model uses only pedigree and phenotype records in the analysis*.

e *The year-class 2016 families were genotyped with 39,189 SNPs. After data QC, we had 34,640 and 1,624 effective SNPs and animals, respectively*.

f *Genetic parameter estimate (± standard error): $\sigma_{a}^{2}$ is the additive genetic variance; $\sigma_{f/d}^{2}$ is the variance due to nested effects of families within challenge date; $\sigma_{e}^{2}$ is the residual error; and h^2^ is the estimated narrow-sense heritability. For the binary survival STATUS, the heritability estimated on the underlying scale of liability was transformed to the observed scale of disease survival*.

g *This total of n = 1,624 fish included the genotyped training (n = 1,449 offspring fish), testing (n = 124 offspring fish) and parents (n = 51 sires) samples*.

### Estimation of Genetic Parameters for IHNV Resistance

The binary survival STATUS records (*n* = 5,191) were fitted to an animal threshold model to estimate genetic variance parameters for IHNV resistance. The variance components analysis was conducted using pedigree-based BLUP (PBLUP) and PBLUP with genomic information (ssGBLUP) under a Bayesian framework, using computer applications from the software BLUPF90 (35). The binary records of survival STATUS were analyzed using an animal threshold model with the software THRGIBBS1F90. We utilized the same statistical model described below in the section of GS analysis with single-step GBLUP. The Gibbs sampler collected data from a total of one million iterations, of which the first 200,000 iterations were discarded; one sample was saved from every 100 iterations from the remaining 800,000 iterations; thus, results from 8,000 independent samples were used in the analysis. The proper mixing and convergence of the Markov chain Monte Carlo (MCMC) iterations were assessed with an script written using the R package CODA (38).

The heritability for survival STATUS was calculated as: $h^{2}=\sigma_{a}^{2}/\left(\sigma_{a}^{2}+\sigma_{f/d}^{2}+\sigma_{e}^{2}\right)$; where *h*^2^ is the narrow-sense heritability; $\sigma_{a}^{2}$ is the additive genetic variance; $\sigma_{f/d}^{2}$ is the variance due to the nested effect of families within the challenge date; and $\sigma_{e}^{2}$ is the residual error variance. The heritability for the binary survival STATUS estimated on the underlying scale of liability using a threshold model was transformed to the observed scale of disease survival STATUS using methods described elsewhere (23).

### GS Using Single-Step GBLUP Methods

The marker genotype data from training fish and pedigree information on all fish included in this GS study were used to estimate GEBV for the genotyped testing fish sample (*n* = 124) using two methods: (i) ssGBLUP (17, 39); and (ii) wssGBLUP (40). These single-step methods use all available information on sampled fish, including pedigree, genotype, and phenotype records, as well as those offspring fish without genotype data, *n* = 3,742 (17, 39). The CSF sample used in this GS study included *n* = 5,191 offspring fish from 104 YC 2016 families that had IHNV resistance data (Table 1). From these 5,191 phenotyped offspring fish, a subset of 1,449 had genotype data from 34,640 effective SNPs (Table 2).

In GS with ssGBLUP, the weight for each SNP is 1 for the first iteration, which means that each SNP has the same weight (i.e., single-step GBLUP). For the following iterations (2nd, 3rd, etc.), the weights are SNP-specific variances that are calculated using the estimate of the SNP allele-substitution effect from the previous iteration and the corresponding SNP allele frequencies (24). Estimates of SNP effects were computed using a weighted relationship matrix, using this equation: $\hat{\text{u}}={\text{D}\text{M}}^{\prime }{\left[{\text{MDM}}^{\prime }\right]}^{-1}{\hat{\text{a}}}_{\text{g}}$, where $\hat{\text{u}}\;$ is the vector of the estimated SNP effects; **D** is a diagonal matrix of weights for variances of SNP effects; **M** is a matrix relating genotypes of each SNP to each individual; and ${\hat{\text{a}}}_{\text{g}}\;$is the estimate of the additive genetic effect for genotyped animals. The individual variance of SNP effects, which corresponds to the diagonal elements of **D**, was estimated as (41): ${\hat{\sigma }}_{u,i}^{2}={\hat{u}}_{i}^{2}2p_{i}\left(1-p_{i}\right)$, where: ${\hat{u}}_{i}^{2}$ is the square of the effect at SNP **i**, and **p**_**i**_ is the observed allele frequency for the second allele of SNP **i**. In this GS study, we used results from the 1st (ssGBLUP) and the 2nd iteration (wssGBLUP), because usually the 2nd iteration estimates genomic predictions (32) and SNP effects (24, 42, 43) with the highest accuracy.

We fitted a threshold mixed model for the binary data survival STATUS using this animal model: **y=1**μ**+Za+Wc+e**, where **1** is a vector of 1s, μ is the overall mean of phenotypic records, **a** is a vector of random individual animal effects, **c** is a vector of random common environment effects, **e** is a vector of residual effects, and **Z** and **W** are incidence matrices relating records to random animal and common environment effects in **a** and **c**, respectively. The variances of **a**, **c** and **e** are:

$$\begin{array}{l} var\left[\begin{array}{c} \text{a}\\ \text{c}\\ \text{e} \end{array}\right]=\left[\begin{array}{ccc} \text{H}\sigma_{a}^{2} & 0 & 0\\ 0 & \text{I}\sigma_{c}^{2} & 0\\ 0 & 0 & \text{I}\sigma_{e}^{2}\; \end{array}\right], \end{array}$$

where $\sigma_{a}^{2}$, $\sigma_{c}^{2}$ and $\sigma_{e}^{2}$ are additive genetic, common environment and residual variances, respectively, and **H** is a matrix that combines pedigree (**A**) and genomic (**G**) relationship matrices, as in Aguilar et al. (17), and its inverse as defined elsewhere (17, 39). The fish offspring from each FS family were assigned to one tank for IHNV challenge evaluation, so the tank and family effects were confounded. The 100 tested families were evaluated in 11 challenge dates (date), with 7–10 families per date. This nested random family/date effect was used to account for the common environment effect.

The GS for the binary survival STATUS was conducted using Bayesian methods implemented in the software BLUPF90 (35). Exactly, the GS for STATUS with ssGBLUP and wssGBLUP were conducted using the computer program THRGIBBS1F90. The Gibbs sampling scheme and the methods used to assess the correct mixing and convergence of the MCMC iterations were similar to those described in the section of estimation of genetic parameters for IHNV resistance.

### GS Using Single-Step Bayesian Multiple Regression

We conducted GP for the binary IHNV survival STATUS with a single-step Bayesian multiple regression (ssBMR) method using 1-Mb non-overlapping SNP windows (25, 27). The ssBMR method uses the pedigree information and all fish that had phenotype and genotype records, as in the wssGBLUP method. In ssBMR, the genotypes for non-genotyped animals are imputed explicitly given the pedigree data, and corresponding imputation residuals are fitted in the ssBMR model. Thus, the GP with ssBMR model was conducted using the same phenotype and genotype records used for wssGBLUP (Tables 1, 2).

We fitted a threshold mixed model for the binary data STATUS using this animal model: **y=1**μ**+Xb+Z**_**ϵ**_**ϵ+Za+Wc+e**; where **X** is an *n***×***k* matrix of observed or imputed genotype covariates for *k* total number of SNPs across the genome for both genotyped and non-genotyped *n* individuals; **b** is a vector of *k* additive SNP effects; **ϵ** is the imputation residuals for non-genotyped individuals, **Z**_**ϵ**_ is the design matrix allocating records to breeding values of non-genotyped individuals; and **a** is a vector of random polygenic effects. The other elements of the model were already described in the section of GS with ssGBLUP. Scaled inverse chi-squared distributions were used for genetic variance and residual variance as described in (25). In these priors, the degree of freedom was 4, and scaled parameters were estimated by assuming the proportion of variance of the phenotypic data explained by the regression is 0.5. The GS analysis for the binary STATUS was performed with ssBMR using the Bayesian variable selection methods BayesB and BayesC (26) implemented in the software JWAS (44).

In the BayesB and BayesC, the prior assumption is that the marker effects have identical and independent mixture distributions (21). So, both methods fit a mixture model to estimate marker effects (21), which assume that there are two types of SNPs: a fraction (1−π) of SNPs with non-zero effects and another fraction (π) of SNPs that a-priori have zero effect on the quantitative trait (45). In BayesB, the variance parameter assumed for the random SNP effects is specific to each fitted locus (46). On the contrary, an effect variance that is common to all SNPs is used in BayesC (47). In this study, the parameter π was treated as unknown with a uniform prior and estimated with BayesBπ and BayesCπ models, separately, using the option “estimatePi=true” in the ssBMR analysis. We also tested the impact of treating π as known and setting it to 0.999 on the GP accuracy when performing ssBMR with BayesB and BayesC models. In a previous study with this dataset, we tested few mixture parameters (π = 0.990, 0.995, 0.999) and found that ssBMR-BayesB with π = 0.999 detected the largest number of genomic windows associated with IHNV resistance and with the largest additive genetic variance (8).

The BayesB and BayesC methods use Gibbs sampling in the GS analysis (46). The characteristics of the Gibbs sampler and diagnosis methods to test the proper mixing and convergence of the MCMC iterations were like those used in the section of estimation of genetic parameters for IHNV resistance.

### Accuracy and Bias of Breeding Value Predictions

The accuracy of predicted breeding values G(EBV) was estimated as the correlation of the mid-parent G(EBV) with the mean progeny performance (MPP) for each progeny tested family (). After data QC, we had disease challenge data from 62 progeny testing families (*n* = 6,198 fish).

The bias of the predicted G(EBV) was estimated as the regression coefficient of MPP on the predicted mid-parent G(EBV) for each PTF. A value of 1.0 for the regression of true BV, performance phenotype or MPP on predicted G(EBV) is theoretically expected for unbiased estimates of G(EBV); and a deviation from 1.0 can be interpreted as prediction bias (48, 49). Before estimating the bias or regression coefficient, the predicted G(EBV) for the binary survival STATUS, which was estimated on the underlying scale of liability, was transformed to the observed scale. The categorical data analysis performed with the software BLUPF90 and JWAS uses a probit link function; consequently, the estimated G(EBV) was transformed to the standard normal cumulative distribution function (CDF) to estimate the probability of survival (50, 51).

### Relatedness Between Training and Testing Animals in GS Studies

We calculated relatedness between training and testing animals using genomic and pedigree data in the current GS study for IHNV resistance. First, the genomic (**G**) and pedigree-based relationship (**A**_**22**_) matrices were calculated using the software BLUPF90 (35); the **A**_**22**_ is a pedigree-based relationship matrix only for genotyped animals (52, 53). Then, we calculated summary statistics (e.g., sum, average, variance, standard deviation, minimum, maximum and total number of relationships) for sub-elements of the **A**_**22**_ and **G** matrices, separately, using a recently developed Fortran90 software which is on request available from the authors.

Likewise, we also computed relatedness between training and testing animals in our already published GS study on resistance to bacterial cold water disease (BCWD) (23) using the above outlined methods. From this published BCWD study, the dataset in which both the training and testing sample sets included siblings from the same 25 families (each with ~40 offspring fish) was used to estimate relatedness between training and testing animals. Subsequently, we compared the relatedness between training and testing animals estimated in the current GS for IHNV resistance study and the previously published GS for BCWD resistance study.

### Linkage Disequilibrium and Effective Population Size Estimates

The linkage disequilibrium (LD) analysis comprised 100 unrelated fish from the YC 2016 families from the CSF commercial breeding population. We developed five-replicated samples each with 100 unrelated individuals by randomly sampling, with replacement, one offspring fish from each of the 100 YC 2016 families. Thus, the LD analysis was performed using data from 100 unrelated fish (*n* = 100) genotyped with 34,640 effective SNPs. It should be noted that the genotype data that we used in the LD analysis was a subset of the training sample that we used for the GP with 100 genotyped YC 2016 families (9–30 offspring per family).

The LD was estimated using described procedures (54). Briefly, the LD was computed as the Pearson's squared correlation coefficient (*r*^2^) for each pair of allele counts at two linked loci on a chromosome, and the *r*^2^ was estimated for adjacent SNPs within each chromosome. The LD was estimated using the computer program PREGSF90 (55) from the software BLUPF90 (35) using the following expression: $r^{2}=\frac{D^{2}}{p_{1}p_{2}q_{1}q_{2}}$; where *D* = *p*_11_*p*_22_ − *p*_12_*p*_21_ corresponded to the frequency of the genotypes; *p* and *q* are the alleles frequencies. The *r*^2^ estimates were adjusted for experimental sample size with this expression: $r_{adj}^{2}=r^{2}-{\left(2n\right)}^{-1}$, where *n* is the number of unrelated individuals used in the LD analysis (56).

The LD decay with physical distance was estimated using already described procedures (54). Briefly, the LD decay with distance between markers was estimated with the Sved's equation (57): $LD_{ij}=\frac{1}{\left(1+kNe_{t}d_{ij}\right)}$; where *LD*_*ij*_ is the estimated LD for the marker-pair *i* and *j; k* is a constant based on the type of chromosome used in the LD analysis (*k* =*4* for autosomes); *N*_*e*_*t*__ is the effective population size for chromosome *t* and *d*_*ij*_ is the distance between markers *i* and *j*. The *N*_*e*_*t*__was calculated using the Saura's expression (56): $Ne_{t}={\left(4d_{t}\right)}^{-1}[{\left(r^{2}{}_{t}-{\left(2n\right)}^{-1}\right)}^{-1}-\alpha ]$; where *d*_*t*_ is the average length of chromosome *t* in Morgan units; $r_{t}^{2}\;$is the average LD of the chromosome *t;* (2*n*)^−1^ is the adjustment term for experimental sample size; and α is a fixed parameter related to mutation (1= absence of mutation; 2= presence of mutation), and we used α = 2.

## Results

### Heritability of IHNV Resistance

The estimates of narrow-sense heritability (*h*^2^) were 0.22 and 0.25 for ssGBLUP and PBLUP models, respectively (Table 2). The estimate of heritability using genomic data (*h*^2^ = 0.22 ± 0.06) was somewhat lower and with smaller standard error than the estimate with the pedigree-based model (*h*^2^ = 0.25 ± 0.12).

### Accuracy and Bias of BV Predictions for IHNV Resistance

**Figure 1:**
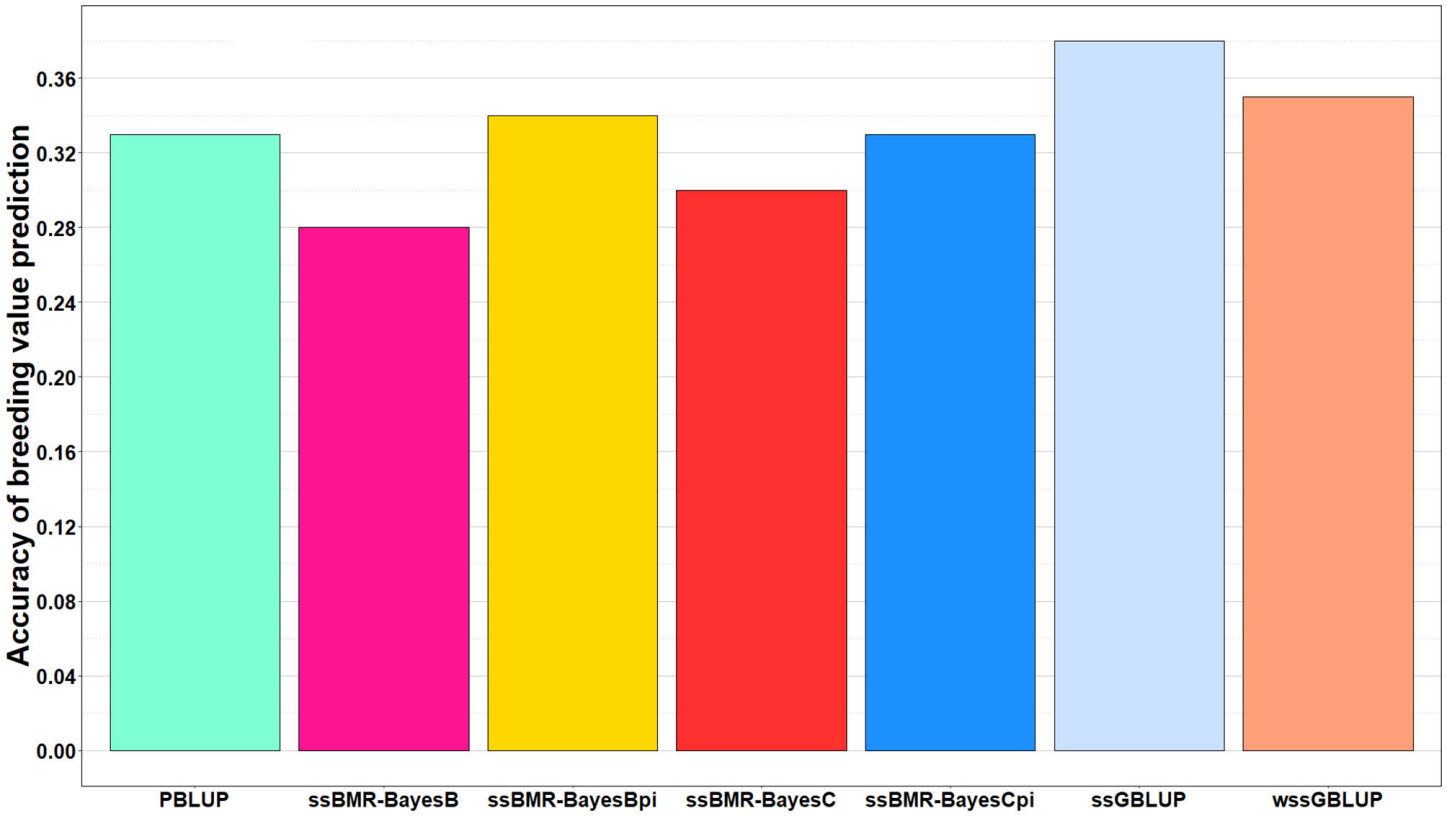
Accuracy of breeding value predictions for IHNV resistance using multiple regression based single-step methods and pedigree-based PBLUP. The genomic breeding value predictions were performed with single-step GBLUP (ssGBLUP), weighted ssGBLUP (wssGBLUP) and four single-step Bayesian multiple regression models (TIFF file).

The accuracy of BV predictions was higher for the GP models ssGBLUP (0.38), wssGBLUP (0.35) and ssBMR-BayesBπ (0.34) than for the pedigree-based PBLUP model (0.33), and the PBLUP and ssBMR-BayesCπ models had similar accuracy (Table 3). However, the PBLUP model (0.33) had higher accuracy GP than the ssBMR models BayesB (0.28) and BayesC (0.30) when using a mixture parameter of π = 0.999. Among the GP models, both single-step GBLUP models (ssGBLUP and wssGBLUP) had higher accuracy of GP than the four tested ssBMR models (Figure 1). Among the ssBMR models, the BayesBπ and BayesCπ (0.33–0.34) had higher GP accuracy than the BayesB and BayesC (0.28–0.30) when using a mixture parameter of π = 0.999 (Table 3 and Additional Table S3).

**Table 3:** Accuracy and bias of breeding value predictions for IHNV resistance^a^.

**Model^b^**	**Accuracy^e^**	**Bias^f^**	**Relative increase of GS accuracy over PBLUP (%)^g^**	**Fitted SNPs in ssBMR model^i^**
PBLUP	0.33	0.60	Na^h^	Na^h^
ssGBLUP	0.38	0.58	15.2	Na^h^
wssGBLUP	0.35	0.38	6.1	Na^h^
ssBMR-BayesBπ^c^	0.34	0.53	3.0	741
ssBMR-BayesB^d^	0.28	0.36	−15.2	35
ssBMR-BayesCπ	0.33	0.32	0.0	6,758
ssBMR-BayesC^d^	0.30	0.32	−9.1	35

a *Clear Springs Foods, Inc. year-class 2016 rainbow trout breeding population. The IHNV resistance phenotype: binary fish survival status (STATUS) after IHNV challenge*.

b *The breeding values for IHNV resistance were estimated using these models: pedigree-based BLUP (PBLUP), single-step GBLUP (ssGBLUP), weighted ssGBLUP (wssGBLUP), and single-step Bayesian multiple regression (ssBMR) with BayesB (ssBMR-BayesB) and BayesC (ssBMR-BayesC) models*.

c *The mixture parameter π was assumed unknown and estimated by the ssBMR analysis. The posterior mean for π from the Bayesian analysis with BayesBπ and BayesC was π = 0.979 and π = 0.805, respectively*.

d *The mixture parameter π was assumed known and set to π = 0.999 in the ssBMR analysis*.

e *The accuracy of predicted G(EBV) was defined as the correlation of mid-parent G(EBV) with the mean progeny performance (MPP) from each progeny tested family (PTF)*.

f *The bias of predicted G(EBV) was defined as the regression coefficient of mean progeny performance (MPP) from each progeny tested family (PTF) on predicted mid-parent G(EBV)*.

g *Relative over-performance of the GS model in comparison to the traditional pedigree-based PBLUP model*.

h *Na indicates that these data cells are not available*.

i *The number of SNPs with non-zero effect on IHNV resistance (k) that are fitted in the ssBMR model at each iteration was estimated with this expression: k = (1 − π)p; the analysis was performed using p = 34,640 effective SNPs*.

**Figure 2:**
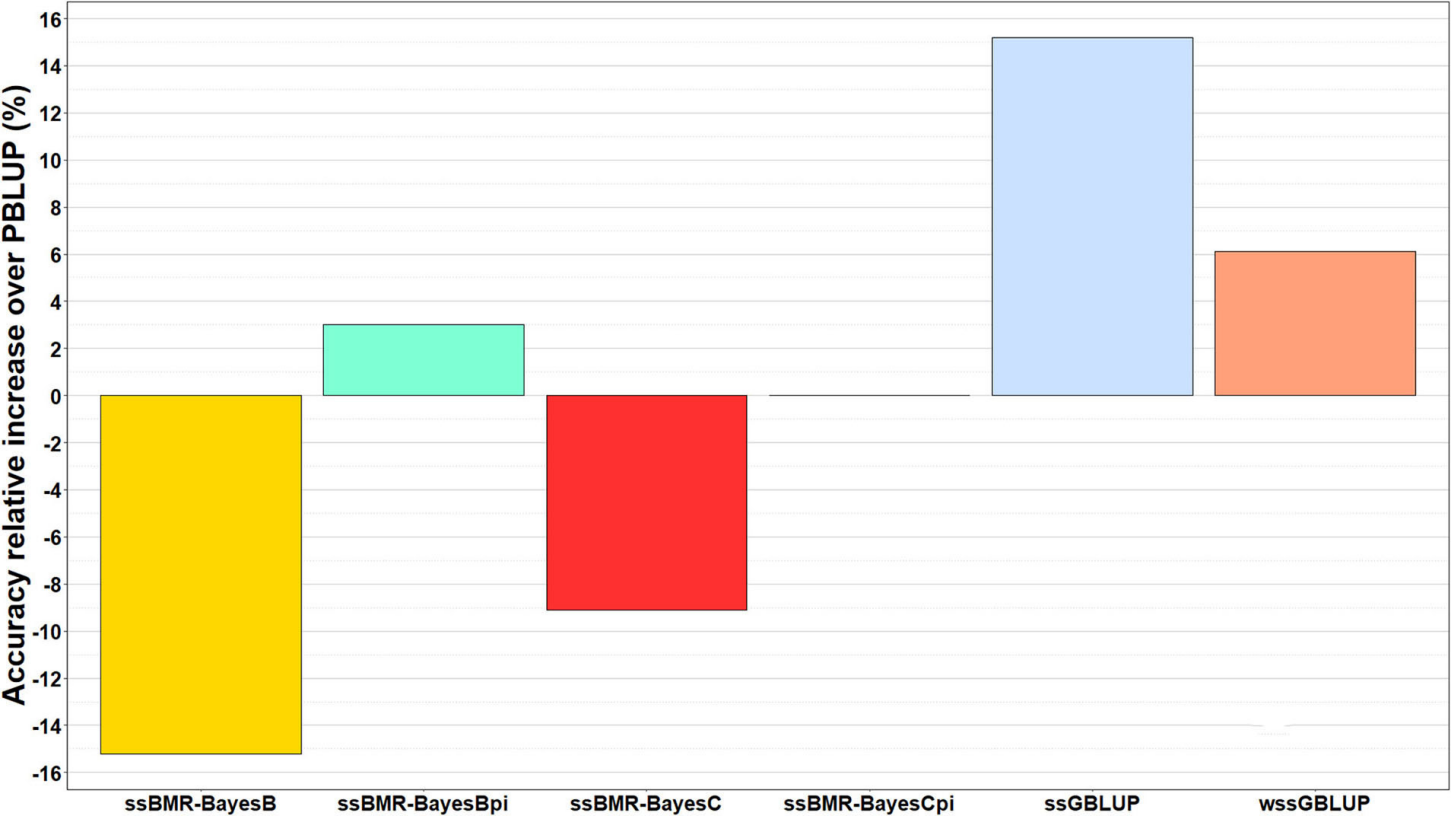
Relative increase in accuracy of genomic predictions over those estimated with pedigree-based PBLUP model. The genomic breeding value predictions were performed with single-step GBLUP (ssGBLUP), weighted ssGBLUP (wssGBLUP) and four single-step Bayesian multiple regression models (TIFF file).

The relative change in accuracy of GP methods compared to the pedigree-based PBLUP model is shown in Figure 2. The accuracy of predictions with the ssGBLUP, wssGBLUP and ssBMR-BayesBπ models outperformed the PBLUP model, by 15, 6, and 3%, respectively (Table 3). Conversely, the accuracy of predictions with PBLUP outperformed the BayesB and BayesC models when using a mixture parameter of π = 0.999.

The bias of BV predictions was lower (i.e., closer to 1.0) for PBLUP (0.60), ssGBLUP (0.58) and ssBMR-BayesBπ (0.53) than for wssGBLUP (0.38) and ssBMR-BayesB (0.36) (Table 3). The ssBMR-BayesC and ssBMR-BayesCπ models had the most biased GP (0.32). Overall, the predicted breeding values for IHNV resistance were biased downwards in this study (i.e., average bias of 0.44).

### Estimates of Relatedness Between Training and Testing Animals

To assess why the accuracy of genomic BV predictions in this study was lower than in our previous published study on resistance to bacterial cold water disease (BCWD) (23), we quantified the level of relatedness between the training and testing fish in both studies. The summary of the genomic (**G**) and pedigree-based (**A**_**22**_) measures of relatedness between training and testing animals calculated in the GS studies for IHNV resistance and BCWD resistance are presented in Table 4. Although the number of effective or high quality polymorphic SNPs was similar in both GS studies (~35K SNPs), the number of total genotyped animals (training and testing fish) was higher in the BCWD study (*n* = 1, 900) (23) than in the current IHNV study (*n* = 1, 573). However, the number of genotyped training animals with phenotypes was higher in the IHNV study (*n* = 1, 449) than in the BCWD study (*n* = 979) (Table 4).

**Table 4:** Genomic and pedigree-based relationship between training and testing animals in genomic selection studies.

**Variable**	**IHNV-CSF 2016^a^**		**BCWD-TLUM 2013^b^**	
	**Genomic (G)**	**Pedigree-Based (A_**22**_)**	**Genomic (G)^d^**	**Pedigree-Based (A_**22**_)**
Training animals (offspring fish)^c^	1,449	1,449	979	1,473
Testing animals (offspring fish)	124	124	921	930
Effective genotyped animals	1,573	Na	1,900	Na
Effective genotyped SNPs	34,623	Na	35075	Na
Sum of relationships	5,415.63	6,226.19	61,634.31	82,841.17
Average of relationships	0.030	0.035	0.068	0.060
Variance of relationships	0.007	0.006	0.011	0.008
Standard deviation of relationships	0.082	0.078	0.105	0.088
Minimum relationship	−0.122	0.000	−0.161	0.000
Maximum relationship	0.697	0.563	0.820	0.531
Total number of relationships	179,676	179,676	901,659	1,369,890

a *Genomic selection study for IHNV resistance in Clear Springs Foods, Inc. (CSF), year-class 2016 rainbow trout breeding population*.

b *Genomic selection study for BCWD resistance in Troutlodge, Inc., all-female, May-spawning (TLUM), year-class 2013 rainbow trout breeding population*.

c *The training dataset included genotype data only from offspring fish. The genotype data from parents were not included in the relatedness analysis*.

d *Relatedness between training and testing animals was estimated for the genomic selection Scheme 3 presented in Vallejo et al. (23), in which both the training and testing sample sets included the same 25 families each with ~40 offspring fish*.

The sum of genomic and pedigree-based relationships between training and testing animals was significatively higher in the GS for BCWD resistance (Σ*G*_*ij*_ = 61, 634; Σ*A*_*ij*_ = 82, 841) than in the current GS for IHNV resistance (Σ*G*_*ij*_ = 5, 416; Σ*A*_*ij*_ = 6, 226). Likewise, the average of genomic and pedigree-based relationships between training and testing animals was substantially higher in the BCWD resistance study $\left({\bar{G}}_{ij}=0.068\;;\;{\bar{A}}_{ij}=0.060\right)$ than in this study $\left({\bar{G}}_{ij}=0.030;\;{\bar{A}}_{ij}=0.035\right)$. The maximum of genomic relationships between training and testing animals was higher in the BCWD study (max *G*_*ij*_ = 0.820) than in this study (max *G*_*ij*_ = 0.697). However, the maximum of pedigree-based relationships was slightly higher in this study (max *A*_*ij*_ = 0.563) than in the BCWD study (max *A*_*ij*_ = 0.531).

### Estimates of Linkage Disequilibrium and Effective Population Size

We evaluated the extent of LD and effective population size (*N*_*e*_) in this rainbow trout commercial population to evaluate the impact of these population genetic parameters on the estimated GP accuracy. The mean LD (*r*^2^), distance between analyzed loci-pairs in base pairs (bp), and *N*_*e*_ estimated for each rainbow trout chromosome in the current study for IHNV resistance is presented in Table 5. The estimated whole-genome average LD was *r*^2^ = 0.26. The average LD per chromosome ranged from 0.21 to 0.39, with average strong LD of *r*^2^ ≥ 0.25 on 17 of the 29 chromosomes.

**Table 5:** Linkage disequilibrium and effective population size in rainbow trout^a^.

**Chromosome**	**Number of SNP**	**Length (bp)**	**Mean SNP distance (bp)^b^**	**Mean (*r*^2^)^c^**	**Mean (N_e_)^d^**
1	1,561	80,463,726	51,546	0.29	30
2	1,483	84,556,146	57,017	0.29	29
3	1,303	78,925,913	60,572	0.24	35
4	1,751	84,440,242	48,224	0.23	24
5	2,360	91,752,627	38,878	0.39	4
6	1,440	80,326,334	55,782	0.27	31
7	1,408	78,384,816	55,671	0.29	33
8	1,533	83,354,286	54,373	0.26	32
9	1,271	67,842,687	53,377	0.23	44
10	1,312	69,431,880	52,921	0.27	33
11	1,369	79,446,316	58,032	0.25	35
12	1,503	81,660,641	54,332	0.25	32
13	646	57,022,654	88,270	0.23	61
14	1,274	80,208,004	62,958	0.24	26
15	1,066	60,567,863	56,818	0.25	38
16	1,437	70,578,346	49,115	0.26	42
17	1,278	70,907,611	55,483	0.24	38
18	1,048	58,027,267	55,370	0.22	44
19	1,027	58,695,507	57,152	0.22	49
20	764	40,785,495	53,384	0.24	48
21	802	49,169,528	61,309	0.21	56
22	924	48,276,594	52,247	0.24	60
23	940	47,802,591	50,854	0.27	43
24	773	40,072,015	51,840	0.23	59
25	1,464	81,698,882	55,805	0.24	25
26	556	35,878,529	64,530	0.27	54
27	789	44,808,344	56,791	0.25	49
28	811	40,281,028	49,668	0.27	46
29	747	42,333,531	56,671	0.27	44
Average	1,194	65,093,083	55,827	0.26	39

a *The linkage disequilibrium (LD) analysis included 100 unrelated fish from the year-class (YC) 2016 families from the Clear Springs Foods, Inc. breeding population. We developed five-replicated samples each with 100 unrelated individuals by randomly sampling, with replacement, one offspring from each of the 100 YC 2016 families*.

b *The average distance, in base pairs (bp), between SNP pairs within each chromosome*.

c *The LD analysis was performed using 34,640 effective SNPs, and the LD was defined as the Pearson's squared correlation coefficient (r^2^) for each pair of allele counts at two linked loci on a chromosome. The r^2^ was estimated for adjacent SNPs within each chromosome and here we present the average LD from five replicated samples*.

d *The mean effective population size (N_e_) from five replicated samples*.

Noticeably, chromosome Omy5 had the highest mean LD (*r*^2^ = 0.39) followed by Omy1, Omy2 and Omy7 (*r*^2^ = 0.29) (Table 5). Chromosomes Omy21 (*r*^2^ = 0.21) and Omy18 and Omy19 (*r*^2^ = 0.22) had the lowest mean LD estimates. The average distance between two adjacent SNPs ranged from 38,878 to 88,270 bp per chromosome with a genome-wide average distance of 55,827 bp.

The average effective population size was *N*_*e*_ = 39 (Table 5). Chromosomes Omy13 (*N*_*e*_ = 61) and Omy22 (*N*_*e*_ = 60) had the largest effective population size. Chromosome Omy5 had the smallest effective population size (*N*_*e*_ = 4). Relatively small effective population size was also estimated for chromosomes Omy4, Omy14 and Omy25 (*N*_*e*_ = 24 − 26).

**Figure 3:**
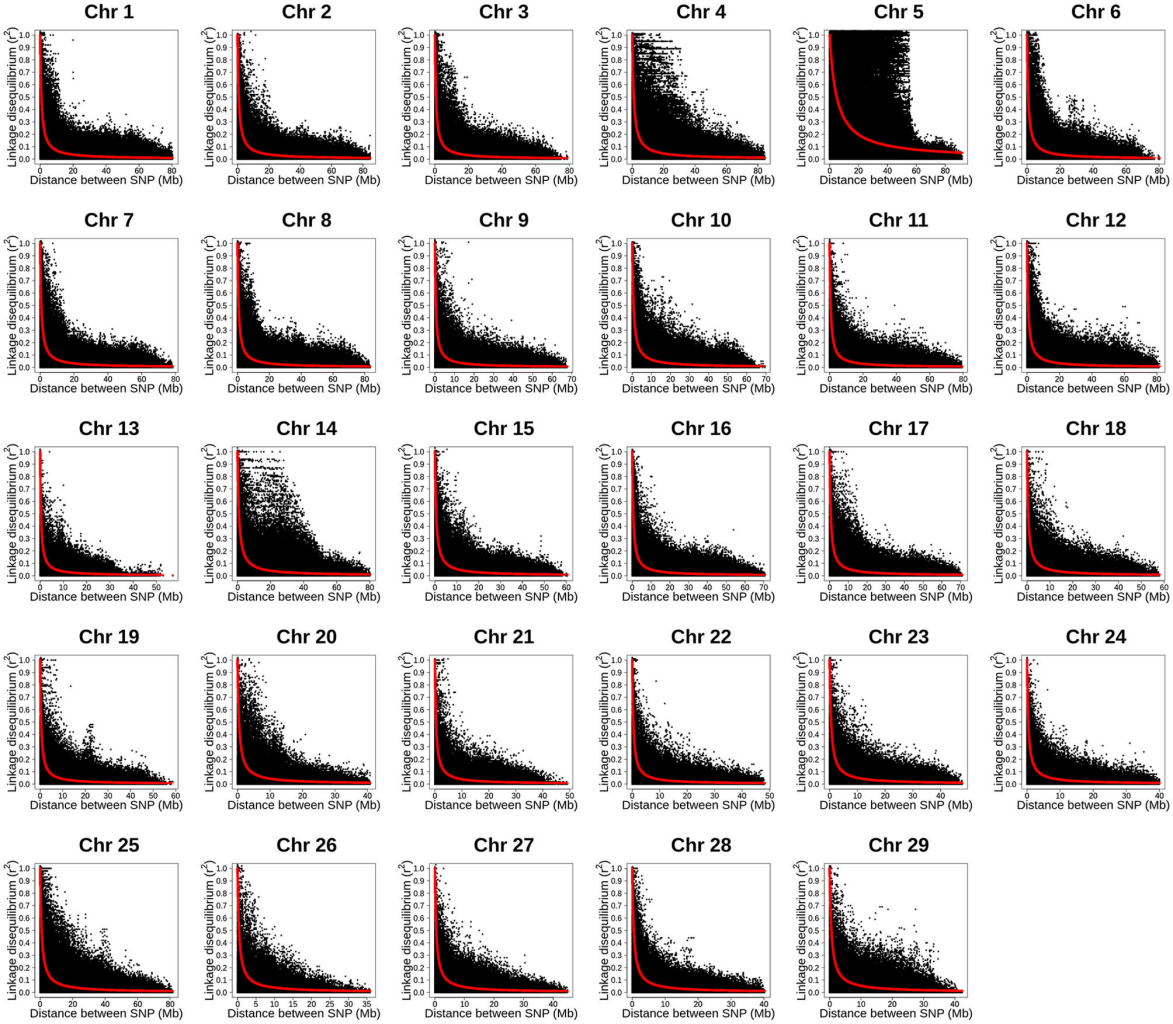
Linkage disequilibrium decay with physical distance estimated by chromosome using 34K SNP panel in year-class 2016 families from Clear Springs Foods, Inc. rainbow trout breeding population. For the LD analysis, in five replicates, one fish from each of the 100 YC 2016 families was randomly sampled with replacement; so, each genotype data set used for LD analysis included 100 unrelated fish. Here, we present results LD decay with physical distance from replication 5 (TIFF file).

The LD decay with physical distance estimated for each of the 29 rainbow trout chromosomes using the ~35K SNP panel are presented in Figure 3. Overall, strong level of LD (*r*^2^ ≥ 0.25) extended over 5 Mb on all the 29 chromosomes. Strikingly, the strong level of LD extended over 40 Mb on chromosome Omy5. In addition, the strong level of LD extended over 20 Mb on chromosomes Omy4, Omy14 and Omy25.

## Discussion

In this study, for the first time using empirical progeny testing data, we found that the gain in GP accuracy achieved with ssGBLUP and wssGBLUP was higher than traditional pedigree-based PBLUP by 15 and 6%, respectively. Thus, these results highlight the potential for effective genetic improvement of resistance against IHNV using genome-enabled selective breeding methods in this rainbow trout breeding population. In addition, our results confirmed the importance of designing GS studies that warrant a high genomic relationship between training and testing animals to estimate high accuracy GP. Furthermore, we found that ssGBLUP had higher accuracy GP than wssGBLUP and ssBMR which suggest that the underlying liability to IHNV resistance might be polygenic. Taken together, these results indicate that whole genome-enabled BV prediction models will be more effective than traditional pedigree-based prediction model or the marker-assisted selection method for improving genetic resistance against IHNV in this commercial rainbow trout population.

### Heritability of IHNV Resistance

The heritability estimates for IHNV resistance in our study were moderate (*h*^2^ = 0.22 − 0.25) (Table 2) and much lower than those reported already using pedigree-based model in a different hatchery rainbow trout population that was naïve to IHNV with no previous history of selective breeding for the trait (2). Our estimates of heritability underline the potential for genetic improvement for IHNV resistance through family-based selective breeding in this commercial rainbow trout population. In this study, the heritability estimate based on genomic data (ssGBLUP) was lower (*h*^2^ = 0.22 ± 0.06) than those based on PBLUP (*h*^2^ = 0.25 ± 0.12). However, the heritability estimated with ssGBLUP had much lower standard error than the heritability estimated with PBLUP.

Strikingly, our estimated heritability for IHNV survival STATUS using genomic data was much lower (*h*^2^ = 0.22 ± 0.06) than the heritability reported by Brieuc et al. (2) for IHNV mortality using a pedigree-based model (*h*^2^ = 0.38; 95*% CI* [0.23, 0.55]). Our studied population has been selected for IHNV resistance for eight generations (Richard Towner, unpublished results) and the population used by Brieuc et al. (2) was not under selective breeding pressure for resistance to IHNV, and therefore had larger genetic variation for IHNV resistance than in this study.

### Accuracy and Bias of Predicted Animal Breeding Values

Recently, we have shown that the ssGBLUP and wssGBLUP had higher accuracy of BV predictions for resistance to IHNV than PBLUP using an stochastic CV analysis (8) and in this study we confirmed those findings using empirical progeny performance records. The estimated GP accuracy for resistance to IHNV was similar between the two studies. In our previous study, the GP accuracy with wssGBLUP (0.39) was higher than with ssGBLUP (0.34), while in the current study the GP accuracy with ssGBLUP (0.38) was higher than wssGBLUP (0.35) (Additional Table S1). The GP accuracy estimated with progeny performance data in this study is more reliable than that estimated with CV analysis (23, 32) because it has been shown that the correlation of mid-parent GEBV with the mean progeny performance for each PTF is a reliable estimator of the accuracy of the predicted breeding values (29, 30).

The accuracy of BV predictions was higher with PBLUP in the present study using progeny performance data (0.33) than in our previous study using CV analysis (0.24) (8). Due to the increased accuracy of predictions with PBLUP in this study, the relative increase in GP accuracy over the pedigree-based PBLUP in this study (6.1–15.2%) was lower than in our previous study (41.7–62.5%) (8). Interestingly, the accuracy of BV predictions for resistance to IHNV we estimated with PBLUP in this study was similar to the accuracy of PBLUP BV predictions we have previously estimated for bacterial cold water disease (BCWD) resistance in a different rainbow trout population (23). However, the estimates of GP accuracy for resistance to IHNV in this study (0.28–0.38) were lower than the accuracy estimates of genomic predictions for BCWD resistance (0.66–0.71) (Additional Table S1). The lower GP accuracy estimated in this IHNV study was due to the sub-optimal GS study design caused by logistical problems and other limitations that were imposed on the study design by the constraints of the ongoing commercial rainbow trout breeding program. One major disadvantage in the IHNV study was that DNA samples and genotype data could not be obtained from weekend mortalities, which together with other missing samples accounted for ~38% of the fish that were phenotyped as dead or susceptible to the disease. While the phenotype records from those fish were available for the PBLUP model, we were unable to obtain genomic information from them, which has substantially reduced the power of the GS models to estimate SNP effects on disease susceptibility. Another major difference was the higher relatedness between the training and testing fish in the BCWD study, which we quantified and later discuss with more detail.

Furthermore, the IHNV and BCWD GS studies differed in these experimental design variables (Additional Table S2): (i) number of phenotyped fish in the training sample (BCWD: *n* = 7,893; IHNV: *n* = 5,191); (ii) sibship size of the training families (BCWD: mean genotyped offspring per family = 29, range 19–40; IHNV: mean genotyped offspring per family = 14; range 9–30); (iii) sibship size of the testing families (BCWD: mean genotyped offspring per family = 31, range 1–44; IHNV: mean genotyped offspring per family = 4, range 1–8); and (iv) number of genotyped fish in testing sample (BCWD: *n* = 930; IHNV: *n* = 124). Consequently, the GS for BCWD resistance afforded higher statistical power to detect marker effects than the GS for IHNV resistance. In addition, another major factor which might have determined the noticeable difference on GP accuracy between these two GS studies was the genetic architecture of the studied traits. The genetic resistance against IHNV might be polygenic in the CSF 2016 population, and thus may require larger size of training sample for high accuracy GP (Daniela Lourenco, personal communication), whereas the genetic resistance against BCWD had an oligogenic architecture in the studied population. Previously, we have found that oligogenic trait architecture is advantageous for estimating high accuracy GP using relatively small-moderate training sample size (23).

The bias of BV predictions for the binary survival STATUS (i.e., IHNV resistance) estimated with CV analysis is incorrect and, consequently, was the most biased (i.e., bias farther from 1.0) (Additional Table S1) (8) due to extreme-phenotype problems in which all binary observations within class variables are identical (i.e., each individual has one binary survival phenotype record of either 0 or 1) (31). In this study, the above problem was avoided by estimating the accuracy and bias of BV predictions using empirical progeny testing data (i.e., offspring survival rate per IHNV challenged progeny testing family) (23, 32). Thus, the BV predictions for IHNV resistance in this study had much lower bias (i.e., bias closer to 1.0) than those estimated with CV analysis (i.e., bias farther from 1.0). However, the bias estimates of predictions in this study were higher than those in a GS study for BCWD resistance (i.e., bias closer to 1.0) conducted in a different rainbow trout population (Additional Table S1) (23).

Overall, the gain in GP accuracy obtained with ssGBLUP and wssGBLUP was 15 and 6%, respectively, higher than using traditional pedigree-based PBLUP. Thus, these results highlight the potential for effective genetic improvement of IHNV genetic resistance in this rainbow trout breeding population using genome-enabled selective breeding method.

### Comparison of Multiple Regression Single-Step GS Methods

The usage of accurate statistical methods and computer algorithms is key for elucidating the genetic basis of resistance to complex diseases and GP using GWAS and GS, respectively. In this study, genomic predictions for IHNV resistance were computed using two multiple regression single-step GS methods that calculate the effect of all markers simultaneously, thus accounting for LD between neighboring loci (21, 46, 58). A unique feature of these multiple regression single-step based methods is that they use all available pedigreed animals with genotype and/or phenotype records in the GWAS and GS analysis. Thus, they have higher power of marker effect detection than those methods that do not use a single-step method and test for association using one-marker at a time without accounting for LD between neighboring loci and without using phenotypes on non-genotyped relatives.

Among the GS models, ssGBLUP had the highest GP accuracy for IHNV resistance (Table 3) followed by GS models (wssGBLUP and ssBMR models) expected to be powerful when analyzing traits that are controlled by few moderate-large effect QTL (i.e., oligogenic inheritance trait). In addition, among the ssBMR models, we found that models that fit a higher number of SNPs (BayesBπ and BayesCπ) had higher prediction accuracy than those that fit a lower number of SNPs (BayesB and BayesC) (Additional Table S3). These results suggest that the genetic resistance against IHNV must be more polygenic than oligogenic. In a previous study, we found that IHNV resistance was controlled by few loci with moderate effects (EGV = 2.0–8.8%) and a large-unknown number of minute-effect loci (8), which further support the hypothesis of polygenic inheritance for IHNV resistance in rainbow trout.

The observed difference in GP accuracy between the two single-step based GS methods (ssGBLUP/wssGBLUP and ssBMR) is due to differences in the underlying model assumptions and the genetic architecture of the analyzed trait. In this study, we evaluated GP models that assume a purely polygenic inheritance for IHNV resistance and a normal distribution of the marker effects such as ssGBLUP, and we also evaluated GS methods such as ssBMR and wssGBLUP, which assume that the trait genetic variance is explained by a reduced number of moderate-large effect QTL, instead of purely polygenic inheritance (25, 59, 60). Indeed, the Bayesian variable selection models run with ssBMR was shown more powerful than standard mixed linear GBLUP-based models when the trait under study is controlled by few loci with moderate-large effect and many minute-effect loci, i.e., oligogenic inheritance trait (23, 60, 61). In this study, ssGBLUP had higher GP accuracy than wssGBLUP and Bayesian variable selection models run with ssBMR. In addition, ssBMR models that fit a high number of non-null effect SNPs (BayesBπ with 741 SNP, and BayesCπ with 6,758 SNP) had higher GP accuracy than ssBMR models that fit a low number of SNPs (BayesB and BayesC each with 35 SNP). Thus, these results suggest that the underlying liability of genetic resistance against IHNV might be polygenic, which imply that the heritable component of IHNV resistance is due to thousands of loci each having a minor effect on liability to IHNV resistance.

### Impact of Relatedness Between Training and Testing Animals on GP Accuracy

The most critical factors for estimating high accuracy GP when performing intra-population GS are (i) the extent of LD between markers and QTL; (ii) the training population sample size; and (ii) the degree of relatedness between the training and testing animals (14, 62, 63). In this study, we computed pedigree-based (**A**_**22**_) and genomic (**G**) relationship between training and testing individuals for two GS studies (Table 4), separately, and found that the average and sum of genomic and pedigree-based relationships in the current GS for IHNV resistance was substantially lower than in the GS for BCWD resistance (23). Intuitively, the level of relatedness between training and testing individuals is defined by the GS study design; and clearly, the design features of the GS for BCWD resistance ensured a higher level of relatedness between the training and testing animals than in the GS for IHNV resistance (Additional Table S2). Thus, the lower level of relatedness between the training and testing fish in the current IHNV resistance study likely contributed to the lower GP accuracy estimates in comparison with the BCWD study.

With simulation-based studies using empirical data from two of our GS studies for BCWD resistance using 100 and 138 families from YC 2013 and YC 2015, respectively, from the TLUM population, we determined that we need to evaluate 20–40 FS offspring per training (and testing) family to compute GP with high accuracy (unpublished results). Thus, when performing intra-population GS with rainbow trout, if the breeding goal is to make population-wide inferences on GP, then we should aim to include in the training and testing sample a large collection of families each with small family sibship size, i.e., 150 families each with ~10 offspring. However, if the breeding goal is to make high accuracy GP in a subset of the population (i.e., pre-selected top performance families with candidate breeders for the next generation), then we should include in the training and testing sample a reduced number of families each with a reasonably large family sibship size, i.e., 30–50 families each with 20–40 offspring. Furthermore, we should include full-sibs from the same families in the training and testing sets to maximize the degree of relationship between the training and testing animals, and thus estimate genomic predictions with high accuracy.

### Linkage Disequilibrium and Effective Population Size

In this study, we estimated for the first time genome-wide distribution of LD and its decay with physical distance using a dense 34K SNP panel in the CSF YC 2016 rainbow trout breeding population. The mean LD across the rainbow trout chromosomes was high (*r*^2^ = 0.26). The average LD estimated in this population was high and similar to the average LD estimated in the TLUM population (*r*^2^ = 0.27) (54). Chromosome Omy5 had the highest average LD (*r*^2^ = 0.39) and also the lowest effective population size (*N*_*e*_ = 4). Similarly, Omy5 had also the highest LD (*r*^2^ = 0.44) and lowest effective population size (*N*_*e*_ = 14) in the TLUM population (54). In Omy5, the higher than average LD is probably caused by large chromosomal inversions detected in other studies, which prevented recombination in fish that are heterozygous to the inversion (37). Overall, this CSF rainbow trout population had an average effective population size of *N*_*e*_ = 39 which was smaller than the estimates of *N*_*e*_ = 145 and *N*_*e*_ = 155 in the NCCCWA (64) and TLUM (54) rainbow trout populations, respectively.

We found that the strong LD level (*r*^2^ ≥ 0.25) extended over 5 Mb in all the rainbow trout chromosomes, over 40 Mb on Omy5 and over 20 Mb on Omy4, Omy14, and Omy25 in the CSF YC 2016 rainbow trout population. In the past, we found that the level of strong LD decayed rapidly at distances >2 cM which is equivalent to ~1.2 Mb in the NCCCWA rainbow trout breeding population (64). Recently, we found strong level of LD extending over 1 Mb on all the chromosomes and over 25 Mb on Omy5 in the TLUM rainbow trout population using 34K SNP panel (54). Therefore, our results confirmed that the extent of long-range LD in this commercial rainbow trout population was as high as those observed in two other rainbow trout breeding populations.

Taken together, we found that the current GS for IHNV resistance and the GS for BCWD resistance (23) studies had similar levels of genome-wide average LD (0.26 vs. 0.27) and total samples size of training genotyped fish (1,500 vs. 1,570), although a different samples size of phenotyped fish (5,191 vs. 7,893) (Additional Table S2). However, the average relatedness between the training and testing animals was substantially lower in the current GS for IHNV resistance than in the GS for BCWD resistance with genetic/pedigree relatedness of 0.035 vs. 0.060 and genomic relatedness of 0.030 vs. 0.068 for the IHNV and BCWD studies, respectively (Table 4). Clearly, the level of relatedness between training and testing animals on each GS study was imposed by the study design (Additional Table S2). Therefore, as already suggested for terrestrial livestock species (14, 62, 63), our results confirm the importance of designing optimal GS studies which warrant a high relatedness between training and testing animals to estimate genomic predictions with high accuracy in rainbow trout.

## Conclusion

Our current study on genomic selection for improving resistance to IHNV in rainbow trout using empirical progeny testing data and multiple regression single-step methods found that genomic selection models improved the accuracy of breeding value predictions by 3–15% over the pedigree-based PBLUP. Furthermore, we found that ssGBLUP had higher GP accuracy than wssGBLUP and ssBMR models which suggest that the underlying liability of genetic resistance against IHNV in this rainbow trout breeding population might be polygenic. Therefore, our results highlight that the GP models will be more effective than either the traditional pedigree-based PBLUP model or the marker-assisted selection method for improving genetic resistance against IHNV in rainbow trout aquaculture.

## Data Availability Statement

The original contributions presented in the study are included in the article/Supplementary Materials, further inquiries can be directed to the corresponding author/s.

## Ethics Statement

Ethical review and approval was not required for the animal study because this study used rainbow trout fin clips collected after controlled exposure to IHNV as part of a selective breeding program at the CSF research facility. As farm animals used in a commercial breeding program, these fish are exempted from regulation under the U.S. Animal Welfare Act and therefore not subject to oversight by an Institutional Animal Care and Use Committee or other such ethics committee. This exemption is defined in U.S. Code title 7, chapter 54, section 2,132 g.

## Author Contributions

YP, RV, JM, and RT conceived and planned the study. JM and RT coordinated and supervised the disease challenges and samples collection. RT provided the pedigree and phenotype records and KS and RL processed the samples and identified the fish for genotyping based on pedigree and phenotype records. YP coordinated and supervised samples processing and SNP chip genotyping. GG performed genotype data quality control and bioinformatics filtering and developed a database pipeline to assemble genotype and phenotype records. RV planned and executed the statistical analyses for the genetic and GS data, and wrote the first draft of this manuscript. BF provided support in performing GS with ssGBLUP and wssGBLUP models, and develop a Fortran software (that uses routines from the BLUPF90 software) to compute relatedness between training and testing animals. HC provided support in performing GS with ssBMR models. All authors read and approved the final manuscript.

## Conflict of Interest

RT and JM were employed by the company Clear Springs Foods, Inc. The remaining authors declare that the research was conducted in the absence of any commercial or financial relationships that could be construed as a potential conflict of interest.
